# Factors affecting development of medication-related osteonecrosis of the jaw in cancer patients receiving high-dose bisphosphonate or denosumab therapy: Is tooth extraction a risk factor?

**DOI:** 10.1371/journal.pone.0201343

**Published:** 2018-07-26

**Authors:** Sakiko Soutome, Saki Hayashida, Madoka Funahara, Yuki Sakamoto, Yuka Kojima, Souichi Yanamoto, Masahiro Umeda

**Affiliations:** 1 Perioperative Oral Management Center, Nagasaki University Hospital, Nagasaki, Japan; 2 Department of Clinical Oral Oncology, Nagasaki University Graduate School of Biomedical Sciences, Nagasaki, Japan; 3 Department of Dentistry and Oral Surgery, Kansai Medical University, Osaka, Japan; George Washington University, UNITED STATES

## Abstract

Methods for preventing medication-related osteonecrosis of the jaw (MRONJ) in cancer patients who have received high-dose bisphosphonate (BP) or denosumab (Dmab) have not yet been established. Tooth extraction after starting medication has been believed to be a major risk factor for MRONJ, and therefore this procedure tends to be avoided. This study investigated the risk factors for MRONJ, with a special reference to the correlation between tooth extraction and development of MRONJ. One hundred and thirty-five cancer patients who were administrated high-dose BP or Dmab were enrolled in the study. Demographic factors, general condition, treatment factors, and dental findings were examined retrospectively using medical records and panoramic X-ray findings. The cumulative occurrence rate of MRONJ was calculated using the Kaplan–Meier method, and the correlation between these variables and development of MRONJ was analyzed by univariate and multivariate Cox regression analysis. MRONJ developed in 18 of 135 patients. The 1-, 2-, and 3-year cumulative occurrence rates were 8.6%, 21.5%, and 29.2%, respectively. The duration of medication before first visit to the dental unit and the presence of a tooth with clinical symptoms were significantly correlated with the development of MRONJ. The rate of MRONJ occurrence in patients who had teeth with clinical symptoms, but who did not undergo tooth extraction, became higher 2 years later than that in patients who underwent extraction of teeth with symptoms, although not significant. Early dental examination and effective preventative care to avoid infection/inflammation are important for preventing MRONJ.

## Introduction

Bisphosphonate (BP) and denosumab (Dmab) are bone-modifying agents (BMA) used for managing osteoporosis, skeletal-related events in association with bone metastases from solid tumors, and multiple myeloma. Since BP-associated osteonecrosis of the jaw was first described by Marx in 2003 [1], case reports of medication-related osteonecrosis of the jaw (MRONJ) have increased. In recent years, patients with MRONJ have tended to be treated with surgical therapy [2–9] rather than with conservative therapy [10–12], since some systematic reviews have shown that surgery is superior to conservative therapy [13–15]. We have also reported a multicenter study of 361 patients with propensity score matching analysis, in which we found that the outcome of MRONJ patients undergoing surgery was significantly better than that of those receiving non-surgical treatment [16].

In contrast, risk factors of and methods for preventing MRONJ remain controversial. Some authors have stated that dentoalveolar surgery, including tooth extraction, is a major risk factor for development of MRONJ [17–19]. Position Paper published in Japan [20] has also described that invasive dental procedures should be avoided as much as possible in cancer patients who have been administered high-dose BMA. On the other hand, some investigators have advocated that, rather than tooth extraction per se, pre-existing inflammatory dental disease, such as periodontal disease or periapical pathology, is a risk factor for MRONJ [21].

The purpose of this study was to investigate the factors related to the development of MRONJ in cancer patients who have been administered high-dose BMA, with a view to appropriate prophylaxis for this disease.

## Materials and methods

The study included 135 patients with malignant tumors who had received high-dose BMA therapies (zoledronate 4 mg/4 weeks, or denosumab 120 mg/4 weeks) at Nagasaki University Hospital, between 2011 and July, 2017, or Kansai Medical University Hospital, between 2014 and July, 2017. All patients were referred to the dental unit of the relevant hospital, and underwent panoramic X-ray examinations and dental treatments, including tooth extraction, if needed. Those who were not followed-up for at least 6 months were excluded from the study.

The variables examined were as follows: 1) Demographic factors: age, sex; 2) general condition: diabetes, smoking habits within the past year, administration of corticosteroids, minimum leukocyte count, and minimum serum albumin levels; 3) treatment factors: type of BMA (BP or Dmab) and duration from the start of BMA administration to referral to the dental unit; 4) dental factors: number of residual teeth, clinical symptoms (pain, swelling, redness, or pus discharge) and type of inflammation (endodontic, periodontal, combined endodontic and periodontal, and pericoronitis), abnormal X-ray findings (apical pathology larger than 3 mm, alveolar bone loss of more than 1/2, root remnant, or root fracture), tooth extraction before the start of BMA administration, and tooth extraction after the start of BMA administration; and 5) development of MRONJ. Clinical symptoms and abnormal X-ray findings were examined after finishing tooth extraction and prior to start of BMA administration.

Statistical analyses were performed using SPSS software (version 24.0; Japan IBM Co., Tokyo, Japan). The cumulative occurrence rate of MRONJ was calculated using the Kaplan–Meier method, and was analyzed by univariate and multivariate Cox regression analysis.

Ethics approval for the study was obtained from institutional review board (IRB) of Nagasaki University Hospital. This was retrospective study, and therefore we published research plan and guaranteed opt-out opportunity by the homepage of our hospital according to instruction of IRB.

## Results

Table 1 shows the background factors of the patients. Sixty-four patients were males and 71 females, with an average age of 62.6 years. Eighty-nine patients (65.9%) were referred to the dental unit prior to the start of administration of BMA, and among them, 39 underwent tooth extraction before administration of BMA, while 46 patients were referred at 0–4061 days (median; 286 days) after the start of BMA therapy. Abnormal panoramic X-ray findings were detected in 56 patients, and clinical symptoms was observed in 25 patients (endodontic in 12 patients, periodontal in 10, combined endodontic and periodontal in 2 and pericoronitis in 1).

**Table 1:** Background factors of the patients.

Factors	Category	MRONJ(-)	MRONJ(+)
Age (years)		61.0±11.8	62.8±11.3
Gender	Male	62	2
	Female	55	16
Sort of BMA	BP	69	11
	Dmab	48	7
First visit to the dental unit	Before BMA administration	83	6
	After MBA administration	34	12
Period from start of BMA administration to first visit to dental unit (days)		153±504	550±884
	< 6 months	101	9
	≥ 6 months	16	9
Smoking habit within one year	(-)	76	15
	(+)	26	1
	Unknown	15	2
Diabetes	(-)	106	17
	(+)	11	1
Steroid administration	(-)	101	16
	(+)	16	2
Minimum leukocytes count (/μL)		2908±1848	2706±1325
Minimum serum albumin value (mg/dL)		2.95±0.707	3.17±0.609
Number of teeth		21.8±7.77	21.2±6.72
Tooth with clinical infection symptom	(-)	102	8
	(+)	15	10
	Endodontic	5	7
	Periodontal	7	3
	Combined endodontic-periodontal	2	0
	Pericoronitis	1	0
Tooth with X-ray abnormality	(-)	72	7
	(+)	45	11
Tooth extraction before start of BMA administration	(-)	83	13
	(+)	34	5
Tooth extraction after start of BMA administration	(-)	102	12
	(+)	15	6
Total		117	18

Tooth extraction after administration of BMA was performed in 20 patients. In most patients, oral penicillin was administrated 1 hour before tooth extraction and for 2 or 3 days after extraction. Eleven patients underwent extraction of teeth with symptoms, and five developed MORNJ. Nine patients underwent extraction of teeth with X-ray abnormality but without symptoms, and none developed MRONJ (Table 2).

**Table 2:** Cause of tooth extraction and development of MRONJ.

Cause of tooth extraction	Number of patients	Development of MRONJ
Clinical infection symptom	11	5
Endodontic	8	4
Periodontal	3	1
Combined endodontic and periodontal	0	0
Pericoronitis	0	0
X-ray abnormality without infection symptom	9	0
Endodontic	1	0
Periodontal	4	0
Root remnant	3	0
Impacted wisdom tooth	1	0
Total	20	5

**Fig 1:**
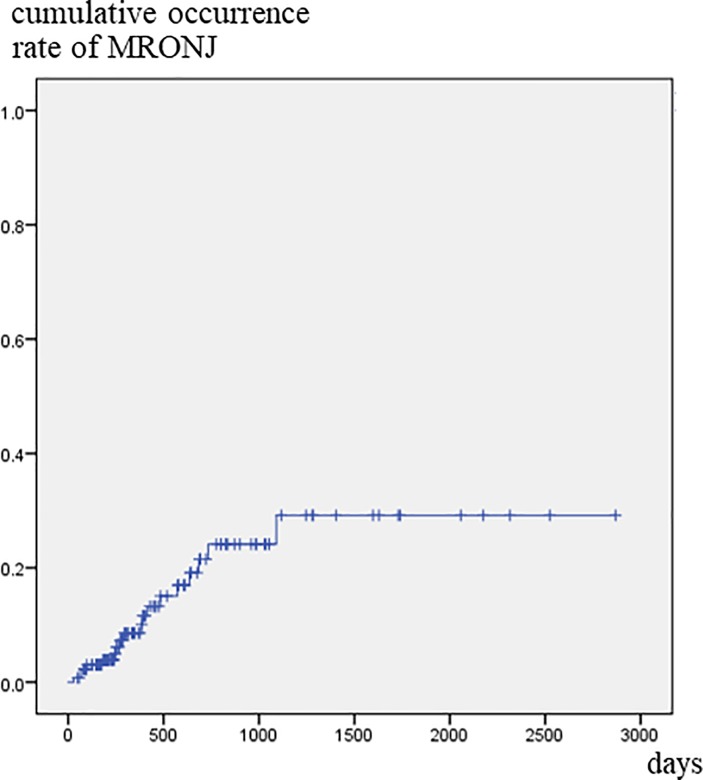
Cumulative occurrence rate of MRONJ. The 3-year cumulative occurrence rate was 29.2% in all 135 patients.

MRONJ developed in 18 of 135 patients. The crude occurrence rate was 13.3%, and the 1-, 2-, and 3-year cumulative occurrence rate was 8.6%, 21.5%, and 29.2%, respectively (Fig 1). Univariate Cox regression analysis revealed that gender, timing of first visit to the dental unit, and tooth with symptoms were significantly correlated with development of MRONJ. By multivariate Cox regression analysis, female (*p* = 0.033), BMA administration for more than 6 month before first visit to the dental unit (*p* = 0.027), and the presence of a tooth with clinical symptoms (*p* = 0.001) were significantly correlated with development of MRONJ (Table 3). Tooth extraction after the start of administration of BMA was not a risk factor. There were no relationship between type of inflammation and development of MRONJ.

**Table 3:** Variable significantly correlated with development of MRONJ by univariate and multivariate Cox regression.

Factors	Univariate Cox Regression			Multivariate Cox Regression**		
	p value	HR	95% CI	p value	HR	95% CI
Age (years)	0.559	1.012	0.972–1.054	0.892		
Gender (male vs. female)	0.020*	0.175	0.040–0.764	0.033*	0.192	0.042–1.039
Sort of BMA (Dmab vs. BP)	0.679	1.224	0.471–3.180	0.297		
First visit to the dental unit (after administration vs. before administration)	0.012*	3.535	1.323–9.445	0.090		
Period from start of BMA administration to first visit to the dental unit (days)	0.030*	1.000	1.000–1.001	0.120		
Period from start of BMA administration to first visit to the dental unit (≥ 6 months vs. < 6 months)	0.001*	5.021	1.986–12.696	0.027*	3.456	1.148–10.402
Tooth extraction before start of BMA administration (+ vs. -)	0.991	0.994	0.354–2.793	0.202		
Smoking habit within one year (+ vs. -)	0.137	0.215	0.028–1.631	0.429		
Diabetes (+ vs. -)	0.586	0.574	0.078–4.230	0.389		
Steroid administration (+ vs. -)	0.865	0.880	0.202–3.837	0.309		
Minimum leukocytes count (/μL)	0.543	1.000	1.000–1.000	0.091		
Minimum serum albumin value (mg/dL)	0.656	1.187	0.559–2.519	0.581		
Number of teeth	0.755	0.990	0.931–1.054	0.579		
Tooth with clinical infection symptoms (+ vs. -)	<0.001*	6.746	2.657–17.124	0.001*	6.647	2.286–19.326
Tooth with X-ray abnormality (+ vs. -)	0.125	2.103	0.814–5.432	0.252		
Tooth extraction after start of BMA administration (+ vs. -)	0.172	1.995	0.741–5.370	0.574		

*significant

**stepwise selection

**Fig 2:**
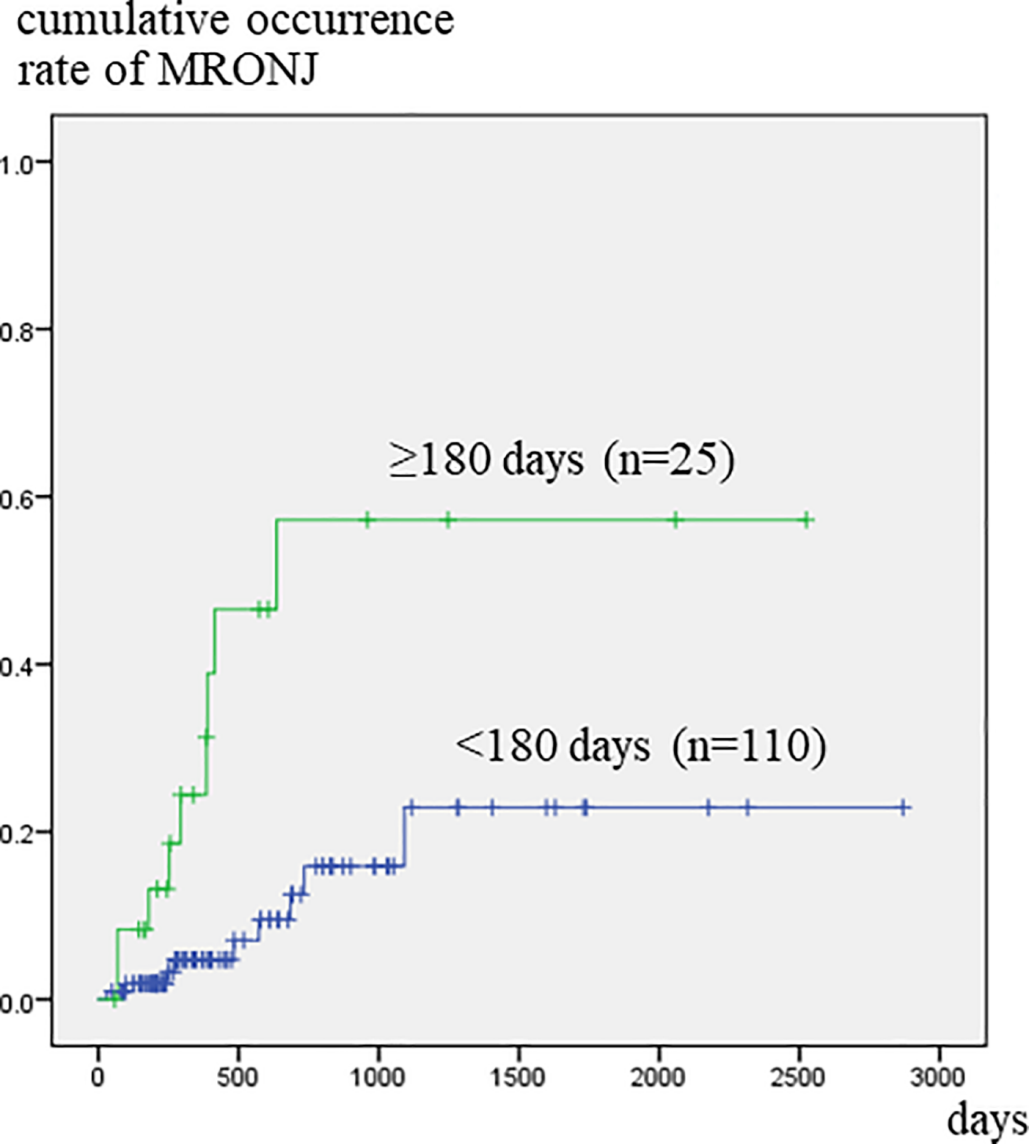
Cumulative occurrence rate of MRONJ according to the time elapsed from BMA administration to referral to dental unit. Patients who were referred to dental unit at more than 180 days after BMA administration show significantly higher occurrence rate.

**Fig 3:**
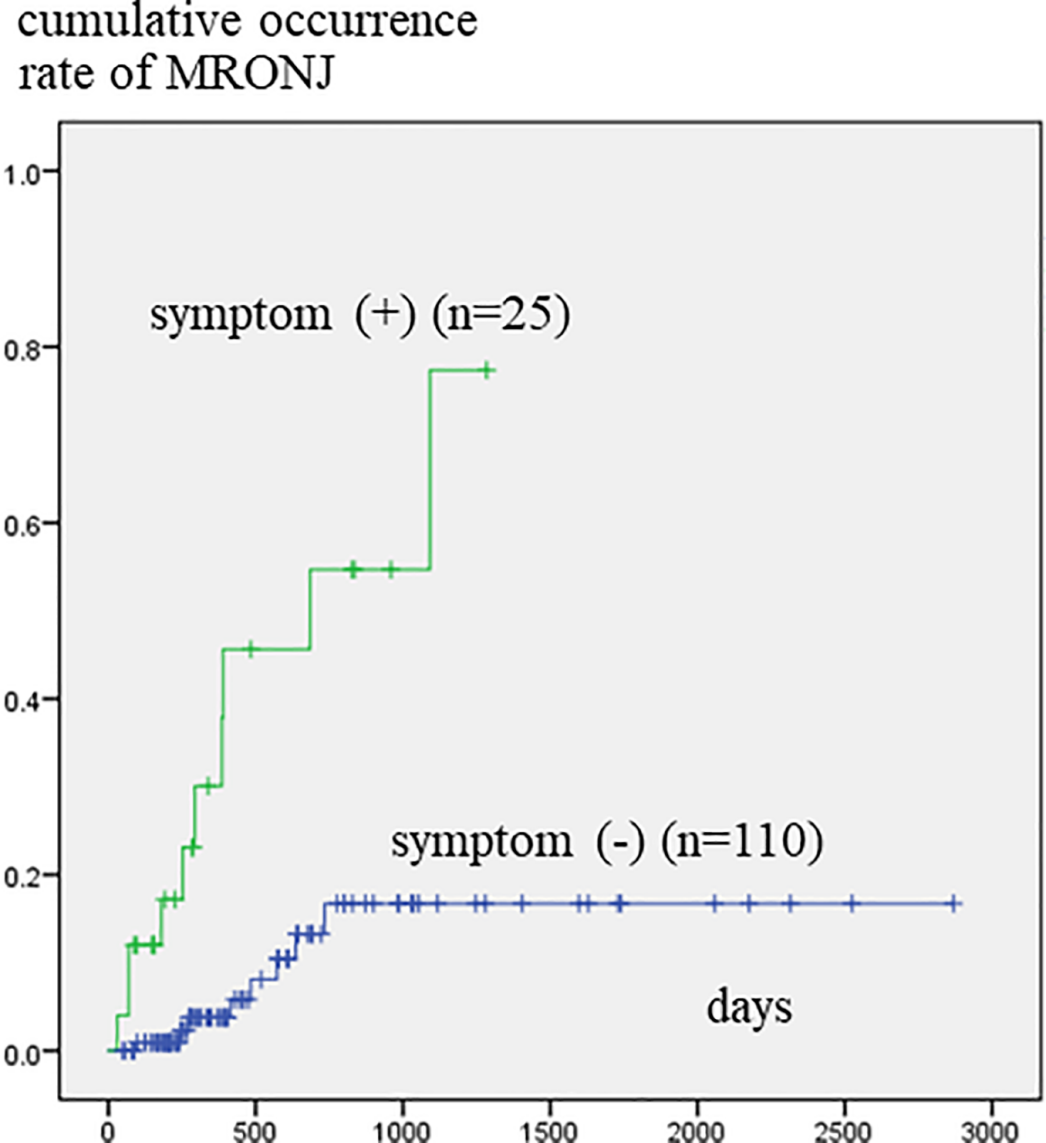
Cumulative occurrence rate of MRONJ according to the presence of clinical symptoms. Patients who had teeth with symptoms showed a significantly higher occurrence rate.

**Fig 4:**
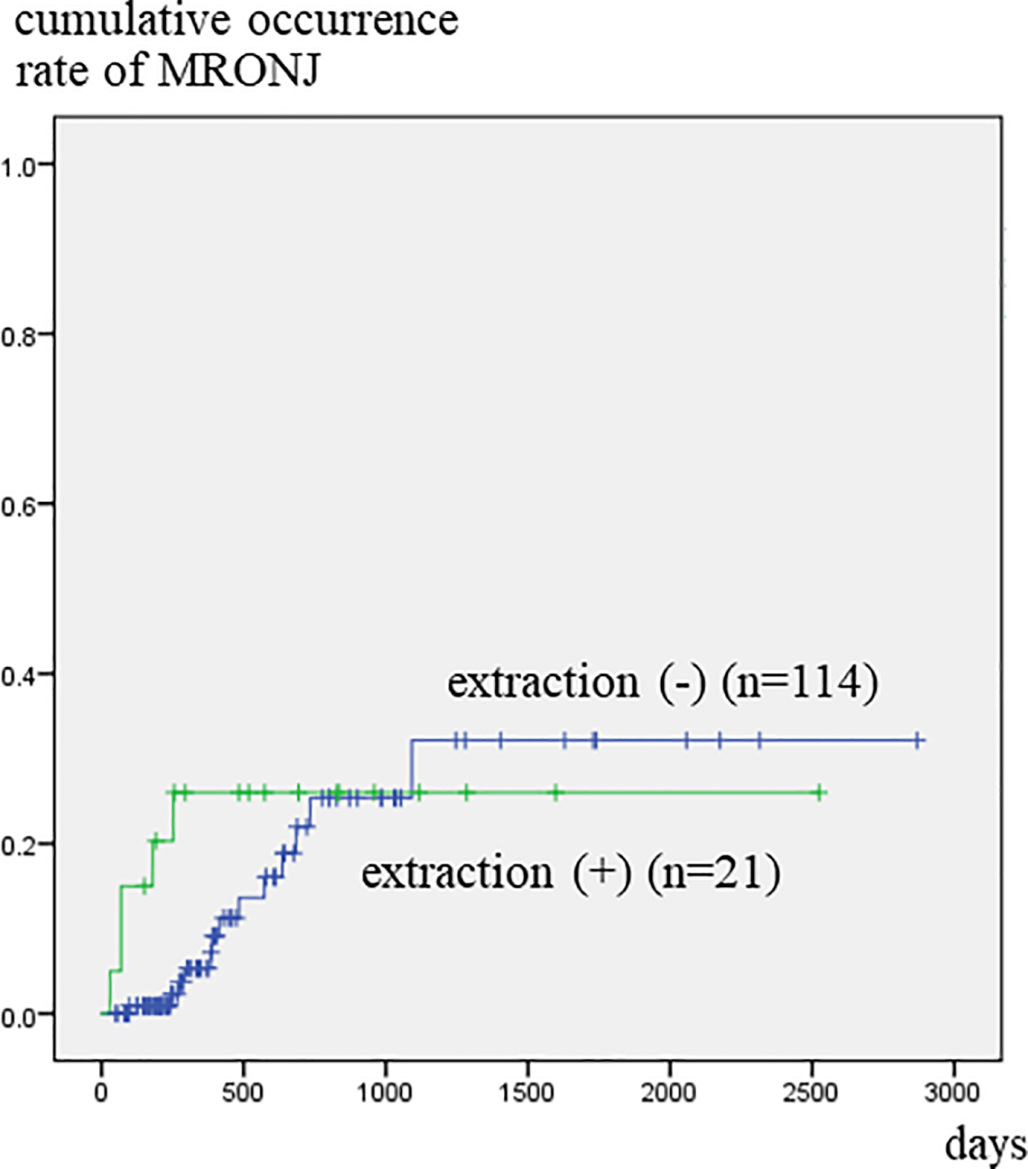
Cumulative occurrence rate of MRONJ according to tooth extraction after BMA therapy. The occurrence rate in patients who did not undergo tooth extraction was low initially, but gradually increased, and exceeded that of patients who underwent tooth extraction 3 years later.

**Fig 5:**
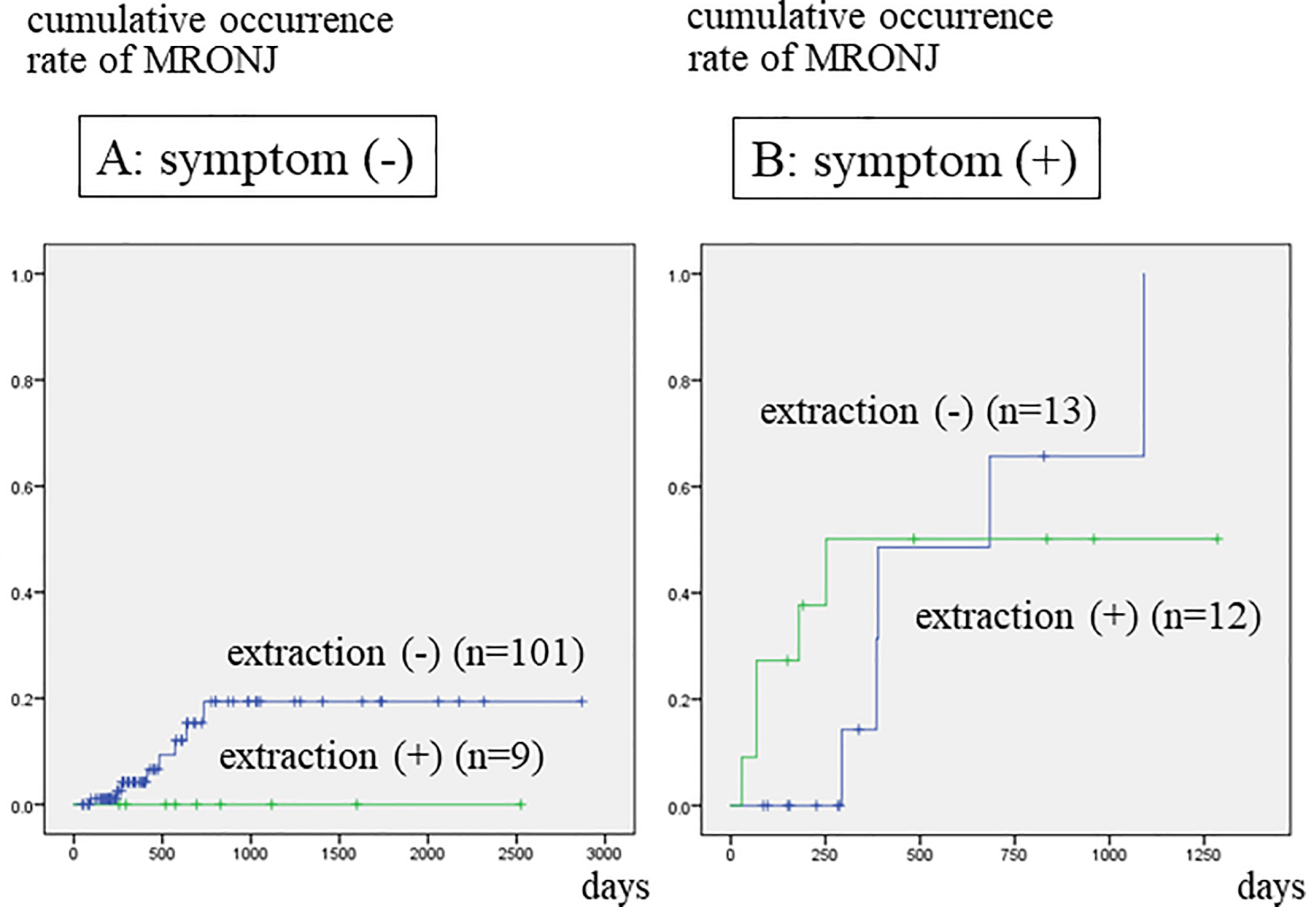
Cumulative occurrence rate of MRONJ according to tooth extraction, after adjustment for pre-existing symptoms. A: Patients who did not have a tooth with clinical symptoms, but who underwent extraction due to abnormalities noted on X-ray imaging, did not develop MRONJ, while those who retained such teeth sometimes developed MRONJ. B: Occurrence rate of MRONJ in patients who had teeth with clinical symptoms, but did not undergo tooth extraction, became higher 2 years later than that in patients who underwent extraction of teeth with clinical symptoms.

Patients who had been referred to a dental unit at more than 180 days after the start of BMA treatment developed MRONJ significantly more frequently than those who were referred before or within 180 days of commencing BMA treatment (Fig 2). Patients that had teeth with clinical symptoms developed MRONJ significantly more often within a short period of time than those with no such teeth (Fig 3). Fig 4 illustrates the relationship between tooth extraction after starting BMA administration and the development of MRONJ. The cumulative occurrence rate of MRONJ in patients who did not undergo tooth extraction was higher than that in patients who underwent tooth extraction after about 3 years. When estimating the association between tooth extraction and development of MRONJ after adjusting for pre-existing symptoms (Fig 5), we found that the rate of MRONJ in patients who had teeth with clinical symptoms, but who did not undergo tooth extraction, became higher 2 years later than those who underwent extraction of teeth with clinical symptoms. On the other hand, patients who did not have teeth with clinical symptoms, but underwent tooth extraction because of abnormal X-ray findings, did not develop MRONJ, while those who retained such teeth sometimes developed MRONJ. In the latter patients, periodontal or endodontic imflammatry lesions appeared and worsened rapidly in spite of usual dental management and developed MRONJ later.

## Discussion

The occurrence rate of MRONJ in patients who received high-dose BMA therapy is relatively low. Out of 5723 patients with malignant tumor, who were administrated high-dose zoledronate or Dmab, MRONJ occurred only in 52 (1.8%) patients who had received zoledronate and 37 (1.3%) with Dmab [22,23]. In contrast, some authors reported a higher occurrence rate; Kajizono et al. stated that 13 of 155 patients (8.4%) administrated zoledronate or Dmab developed MRONJ, and when limited to those with symptom of pus discharge, the incidence was higher (15.6%) [24]. In our multicenter, retrospective study, 11 of 80 cancer patients (15.7%) who were administrated zoledronate or Dmab and underwent tooth extraction developed MRONJ (unpublished data). Furthermore, Fedele et al. [25] reported that 51 of 96 patients (53%) who were administrated BP or Dmab and had clinical symptoms developed MRONJ, and they advocated the concept of stage 0 MRONJ. The discrepancy of the incidence of MRONJ among authors is thought to be due to differences in the inclusion criteria of the patients. Patients with no dental symptoms seem to have a lower risk of MRONJ, even if they receive a high dose of BMA, than those with dental symptoms of infection.

The methods appropriate for treatment of MRONJ, i.e., conservative or surgical treatments, are controversial [2–12]. Although conservative treatment has often been used previously, the opportunity for surgical treatment has recently increased, since some systematic reviews have shown that surgical therapy was superior to conservative therapy [13–15]. In our previous multicenter study, the rate of complete healing of patients administrated BMA and underwent surgical treatment was 76.7%, while that of those undergoing conservative treatment was 25.2%. Furthermore, cancer patients receiving high-dose BMA showed a significantly poorer outcome, i.e., complete healing in 51.5%, when surgical treatment was performed, and 6.9%, when conservative treatment was performed [16]. These figures indicate a worse outcome in those treated with high-dose zoledronate or Dmab, and therefore when high-dose BMA therapy is administered, prevention of MRONJ becomes particularly problematic.

Tooth extraction has been believed to be a major risk factor for the development of MRONJ. Barasch et al. [17] reported that the risk factors of osteonecrosis of the jaw (ONJ) were bisphosphonate use, local suppuration, tooth extraction, and radiation therapy by dentist questionnaires and patients interviews in 191 patients with ONJ and 573 controls. Kyrgidis et al. [18] reported that tooth extraction was associated with a 16-fold increased risk for MRONJ in a case-control study of patients with cancer who were administered zoledronate, while Vahtsevanos et al. [18] stated that tooth extraction was associated with a 33-fold increased risk for MRONJ in a longitudinal cohort study of cancer patients given intravenous BPs. Estimates for development of MRONJ after tooth extraction in patients with cancer exposed to intravenous BPs ranges from 1.6 to 14.8% [26–28].

On the other hand, Otto et al. [21] stated in a report of a retrospective study of 72 patients (27 oral and 45 intravenous BMA) undergoing tooth extraction that it is not the tooth extractions per se, but rather the prevailing infection conditions that may be the key risk factor for the development of MRONJ. The American Association of Oral and Maxillofacial Surgeons (AAOMS) Position Paper on Medication-Related Osteonecrosis of the Jaw (2014 Update) [29] also described that pre-existing dental disease, such as periodontal disease or periapical pathology, may confound the relationship between tooth extraction and the risk for MRONJ, and that it would be valuable to investigate the association between tooth extraction and MRONJ after adjustment for pre-existing inflammatory dental disease.

In the current study, three variables, i.e., gender, duration of medication before referral to the dental unit and the presence of a tooth with clinical symptoms, were correlated with development of MRONJ, according to multivariate analysis, but tooth extraction was not identified as a risk factor. The rate of MRONJ occurring in patients who did not undergo tooth extraction increased gradually, exceeding that of patients who underwent tooth extraction 3 years later. Furthermore, when examining only patients with clinical symptoms, the former rate exceeded the latter rate about 2 year later. These findings suggest that, for patients who are expected to have a long-term survival, it is better to perform early extraction of teeth with symptoms.

This study showed that MRONJ might occur not by tooth extraction but local infection, although it is not clear that the clinical symptom is due to the tooth infection or ONJ itself, that is, stage 0 MRONJ [25]. In fact, it is difficult to distinguish between stage 0 MRONJ and tooth infection. AAOMS [29] described MRONJ stage 0, but International Task Force on Osteonecrosis of the Jaw did not adopt the term of stage 0 [30]. It was not clear whether the infectious symptoms were due to teeth or stage 0 MRONJ, but in any case, MRONJ often developed due to causes other than tooth extraction.

The reason why occurrence rate of MRONJ in female was significantly higher than that in male is not clear. Although smoking is well known to be one of the risk factors for MRONJ, it was not correlated with development of MRONJ in the current study.

Our study had some limitations. First, this was a retrospective study with a small sample size. Second, the presence of clinical symptoms was judged only by subjective symptoms, but detailed objective information, such as pocket depth and bleeding during probing, could not obtained because of the retrospective nature of the study.

In conclusion, it seems likely that it is the underlying infection and not the extraction that puts a patient at increased risk of MRONJ, and an early dental examination and effective preventative care is important to avoid infection and any subsequent requirement for tooth extraction while on antiresorptive therapies. Further prospective clinical trials are needed to determine the most appropriate method for prevention of MRONJ in patients who are administered high-dose BMA.

## Supporting information

S1 Data CRF of 135 patients. (XLSX)
